# The Safety of Probiotics Intended for Use in Pregnant and Lactating Women: From a Desirable to a Required Task

**DOI:** 10.3390/foods13244024

**Published:** 2024-12-12

**Authors:** Leónides Fernández, Belén Orgaz, Juan M. Rodríguez

**Affiliations:** 1 Department of Galenic Pharmacy and Food Technology, Complutense University of Madrid, 28040 Madrid, Spain; leonides@ucm.es (L.F.); belen@ucm.es (B.O.); 2 Instituto Pluridisciplinar, Complutense University of Madrid, 28040 Madrid, Spain; 3 Department of Nutrition and Food Science, Complutense University of Madrid, 28040 Madrid, Spain

**Keywords:** probiotics, safety, pregnancy, lactation, infertility, adverse effects

## Abstract

During pregnancy, women undergo changes that affect virtually every organ, apparatus, or system, including the host microbiota. Most pregnancies progress smoothly despite the common presence of minor side-effects arising from such adaptations. However, some women may experience more serious complications, including gestational diabetes mellitus, preeclampsia, or preterm delivery. Probiotics are one of the products most used to try to prevent or treat any of the minor or severe symptoms or complications that women may experience during pregnancy or lactation; however, most of them have never been tested in such populations and, therefore, their efficacy and safety claims are frequently unsubstantiated. Overall, probiotic trials involving pregnant or lactating women have shown that these products are usually well-tolerated and safe although adverse effects may also exist. Therefore, health professionals attending pregnant or lactating women should be aware of their use and monitor their efficacy and safety. In conclusion, probiotics recommendations for pregnant or lactating women should be based on scientific evidence, opting exclusively for those products that have been designed for the specific target or condition that a pregnant or lactating woman may be experiencing or at risk of, and which efficacy and safety has already been convincingly tested in such populations.

## 1. Introduction

Probiotic microbes have been consumed by humans since at least the Neolithic era, unintentionally at first through fermented foods (e.g., fermented milks) and, more recently, in a growing variety of functional foods and pharmaceutical-like forms (capsules, sachets, drops, ovules, etc.). The long history of safety associated with many of the species included in probiotic products—mainly from the lactic acid bacteria group and the genus *Bifidobacterium*—along with data from in vitro assays, animal studies, and clinical trials, are the main reasons why probiotics are widely assumed to be safe for most populations [1]. However, some adverse effects and risks associated with probiotic intake have also been reported or suggested, including gastrointestinal side effects, systemic infections, negative metabolic and immunological activities, and the potential transfer of harmful genes to members of the host microbiome [2]. It is important to note that the number of documented adverse effects related to probiotic use is very low compared to their long and widespread use.

Pregnant and lactating women are no exception; throughout history and across most cultures, they have consumed probiotic bacteria. In fact, probiotics present an attractive approach to preventing or treating certain health issues or complications that may arise during these life stages. The use of these products has generally proven to be safe in these populations, although, as with other groups, some adverse effects have been reported in a small percentage of clinical trials. Pregnant and lactating women are usually considered vulnerable populations, making the safety of the foods, supplements, or medications they receive particularly important. Therefore, thorough safety assessments should be included in intervention studies involving probiotics targeting pregnant and lactating women.

This article does not aim to be a systematic review or meta-analysis of the safety of probiotics used by these populations but rather to provide a personal perspective from a research group that has participated in clinical trials with probiotics involving pregnant and/or lactating women for the last 30 years. Our impression is that while the use of such products has notably increased among these populations, there has been a corresponding rise in skepticism about the actual usefulness or safety of probiotics among practitioners. This skepticism is primarily based on the low number of well-designed, large-cohort clinical studies addressing their efficacy and safety in these specific populations. In this context, the objective of this article is to show that (a) some specific probiotic strains may be beneficial during these life stages; (b) most studies conducted so far have reported either a lack of adverse effects or the presence of only minor side effects when probiotics are administered to pregnant or lactating women, including those with major pregnancy complications such as gestational diabetes mellitus; and (c) medical confidence in these products may be enhanced by improving safety assessments in clinical trials targeting pregnant and lactating women.

## 2. Adaptations of the Maternal Microbiota During Pregnancy and Lactation

During pregnancy, each women undergoes anatomic and physiologic changes that affect virtually every organ, apparatus, or system. These biological adaptations are required to support and protect the growing embryo and fetus, and to prepare the mother-to-be for the subsequent lactation period. The composition and function of the microbiota also change during this period to potentially contribute to positive or negative pregnancy and lactation outcomes [3,4]. The maternal microbiota has been described as central for reproduction [5] because of the relevant roles that it may play, for example, in the induction of a tolerogenic immune state [6] or in the vertical transmission of the first colonizers of the neonatal gut [7].

In relation to the vaginal microbiota, the high circulating estrogen levels and the lack of menstruation usually lead to a decrease on its variability and diversity by increasing the *Lactobacillus* dominance [8,9,10]. The stability appears to be higher and linked to healthier outcomes when this microbiota is dominated by *Lactobacillus crispatus*. However, diversity may increase during the final trimester of pregnancy, especially when *Lactobacillus iners* is the dominant *Lactobacillus* species [10]. In addition, the risk of yeasts colonization and infection increases as pregnancy progresses because of some pregnancy-related factors, including increased estrogen levels and glycogen production, and decreased cell-mediated immunity [11]. Overall, the composition of the vaginal microbiome may have a strong influence on pregnancy outcomes, including higher or lower risk of miscarriage or preterm delivery [12,13,14].

The gut microbiota undergoes a profound remodeling during pregnancy. Fecal samples from the first trimester are metataxonomically like those of healthy nonpregnant women while, in contrast, samples from the third trimester are similar to those of patients with metabolic syndrome, enhancing the efficiency of energy extraction [3]. The transfer of fecal microbiota from women at the third trimester of pregnancy to germ-free recipient mice can induce some metataxonomic, metabolic, and immunological changes that are commonly observed in metabolic syndrome [3]. In fact, increases in food intake and fat deposition are normal features from early pregnancy onwards while, in mid-to-late gestation some metabolic, hormonal, and immunological changes promote insulin resistance and facilitate lipolysis and gluconeogenesis, providing a continuous supply of glucose to the fetus [4]. Although these diabetogenic-like changes would be associated with a highly detrimental metabolic disease in men and nonpregnant women, such temporary adaptations provide maternal benefits since they are relevant contributions to support fetal growth and to prepare the host for the highly energy-demanding lactation [15,16,17].

Changes in gut microbiota and physiology in late pregnancy (increased intestinal permeability, increased pressure of the gravid womb on gut vessels), highly increase the likelihood of bacterial translocation [4]. This process is coupled with the entero-mammary circulation of immune cells, and seems responsible, at least partly, for generating a specific mammary microbiota during late pregnancy and throughout lactation [18,19,20]. This human milk microbiota is also very relevant to gut colonization during early life and may impact both mammary and infant health [21,22,23,24]. Finally, pregnancy increases susceptibility to a variety of oral diseases, from gingivitis to periodontitis, because of the interrelated hormonal, immunological, and microbiota changes in the oral cavity. In this context, growing evidence suggests that the risk of suffering adverse pregnancy outcomes, from preeclampsia to preterm birth or low birth weight, may be strongly influenced by the composition of the oral microbiome [25,26,27,28].

## 3. Health Problems During Pregnancy and Probiotics

Some of the physiological adaptations from early pregnancy onwards, including those affecting the composition of the microbiome, explain most of the minor problems that may appear during a normal gestation including, among others, gingivitis, nausea, vomiting, bloating, abdominal cramps, constipation, hemorrhoids, fatigue, mood swings, headaches, increased urination, occasional genitourinary infections (urinary tract infections, vaginal candidiasis, and bacterial vaginosis), dizziness, or weight gain [29].

Most pregnancies progress smoothly despite the relatively frequent presence of such usually minor side-effects. However, some women may experience more serious complications that require careful management to ensure a healthy mother and/or fetal outcome. Such complications may arise as an aggravation of a pre-existing disease but, in most cases, they appear for the first time during pregnancy. The most common ones are gestational diabetes mellitus (GDM), preeclampsia, and preterm delivery.

GDM is characterized by hyperglycemia, and it usually resolves after pregnancy. Women with GDM are considered within the risk pregnancy group as this condition is associated with higher rates of preeclampsia and cesarean section, predisposes to macrosomia, and poses an increased risk of metabolic syndrome and type 2 diabetes for the mother–infant dyad later in life. Preeclampsia is a multisystemic complication that is usually diagnosed from the week 20 of gestation and is associated with hypertension and proteinuria. Preeclampsia can affect the placenta, predisposing intrauterine growth restriction or preterm delivery. Premature labor is referred to a delivery that occurs before 37 weeks of gestation, and the risk of short term (sepsis, necrotizing enterocolitis, respiratory or gastrointestinal disorders…) or long term (neurodevelopment) consequences increases as gestational age decreases.

The causes that may lead to pregnancy complications depend on each woman but infectious, inflammatory, immunological, or metabolic-based conditions are usually cited among the main predisposing factors. In turn, such conditions are being increasingly regarded as typical consequences of an altered gut microbiota (the so-called dysbiosis). In the last decades, the microbiota (and, particularly, the gut microbiota) has emerged as one of the main health drivers and, therefore, it has become a good example of an in-fashion field (for better or worse) in biomedicine. Consequently, gut dysbiosis has been associated with a plethora of diseases and disorders, virtually encompassing all medical specialties. Paradoxically, a high percentage of the PubMed articles about gut microbiota are reviews or hypothesis about how to manipulate the gut microbiota to restore an eubiosis state, while only a small number of them deal with original scientific works. This fact raises false expectations among the population, especially in relation to disorders and diseases as complex as autism spectrum disorders, multiple sclerosis, obesity, or different types of cancer [30].

**Figure 1:**
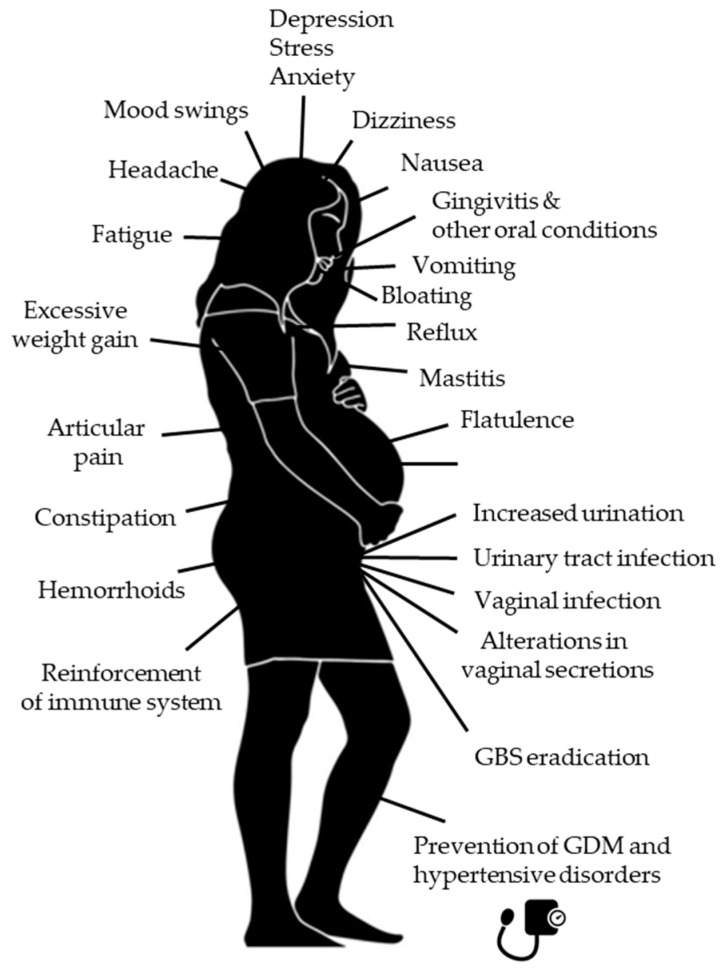
Main minor and severe symptoms or complications of pregnancy and lactation that probiotics claim to prevent or treat, often without any scientific evidence.

In this context, probiotics have been widely marketed as a natural and safe way to restore the microbiota and, thus, to prevent or treat almost any kind of disease. They can be used either by themselves or as an adjunct to conventional therapies. Additionally, they can be found as the so-called functional foods or in a wide spectrum of over-the counter (OTC) pharmaceutical-like presentations, creating a rapidly growing market worldwide. The global market for probiotics reached a value of about 58 billion U.S. dollars in 2022 and was forecast to reach over 85 billion dollars by 2027 [31]. People used to think that OTC are safe just because anyone can buy them, but this is a false perception. As an example, OTC vasoconstrictors containing pseudoephedrine have been linked to a higher risk of heart attack. Anyway, this general perception of safety has made probiotics very popular among general consumers and, also, one of the OTC products most used by pregnant women to try to improve any of the minor or severe symptoms or complications that they may experience during pregnancy [32,33] (Figure 1).

Nowadays, there is an overwhelming abundance of messages in commercials and internet and social networks recommending the use of probiotics in pregnancy because they (sic) “help reduce the symptoms of nausea and vomiting (morning sickness)”, “control blood sugar levels (gestational diabetes)”, “prevent gestational diabetes”, “treat blood pressure and inflammation (preeclampsia)”, “reduce the risk of preeclampsia”, “prevent preterm birth and low birth weight”, “support gastrointestinal and immune health in baby and mom”, “support healthy bowel movements”, “support nutrient absorption”, “support vaginal and urinary tract health” “inhibit the growth of harmful bacteria (bacterial vaginosis)”, “promote postpartum recovery”, “help elevate mood (postpartum depression)”, “reduce symptoms of stress, anxiety, and depression”, “balance mood and mental well-being”, “improve the health status of the mammary gland (mastitis)”, “support immune system form mom and baby”, “minimize antibiotic-associated risks”, “reduce the risk of allergies”, “decrease risk of asthma in children”, “prevent atopic dermatitis (eczema) and food sensitivity in infants”, “decrease fussiness in infants” or provide “digestive harmony” and a “healthy weight gain for mom”. Furthermore, these are just only some of “the extraordinary benefits that probiotics offer to expectant mothers, contributing to both maternal and fetal well-being”. Such recommendations are based on the “multifaceted benefits” observed in “some” studies [34,35].

Although it may seem incredible for both practitioners and consumers, most of these strong health-related claims are made for strains and products which have never been tested in clinical trials and, therefore, there is an absolute lack of scientific evidence backing their use and claims. In addition, the quality control of a large proportion of these products is, at least, highly questionable. In fact, many studies have found relevant discordances between the information provided in the label and their actual content, both in qualitative (absence of publicized strains) and quantitative (presence at much lower concentrations) terms [36,37,38,39,40,41] or the presence of health-inadequate formulations [42]. In addition, even for those strains and products that have been tested in clinical trials, a significant proportion suffer from an incorrect design (including very small cohorts) and under-reported safety [43].

The lack of safety data in health-related products recommended to pregnant women (and their fetuses) is particularly concerning due to their vulnerability and may lead to or contribute to unexpected or undesirable outcomes [33]. As stated above, only very few probiotic strains or products have been tested in pregnant women and, among them, only a few contain a thoughtful report of safety and potential adverse effects, providing details such as type, timing, duration, and severity of symptoms [44].

Many healthcare professionals recognize their lack of solid knowledge on what probiotics are, and do not discriminate among the different products available in the market based on scientific evidence of safety and efficacy for a specific target in a specific population [45,46]. So, while some evidence suggests that a few specific probiotics can be helpful for a few specific pregnancy-related issues, there is a strong and urgent need for improving the quality of evidence, safety reporting, public and professional awareness, and a clear regulation of their use and claims [47].

## 4. Safety of Probiotics During Pregnancy

### 4.1. Generalities

Probiotic-associated risks during pregnancy have been the specific subject of three reviews and two meta-analyses [33,48,49], where information about the target, strains, and posology is provided. They found no mortality or serious adverse effects (AEs) on mothers, fetuses, or infants associated with the intake of these products. Out of 100 studies dealing with the administration of probiotics during pregnancy, only 28 reported AEs and, among these, only 11 studies linked AEs potentially to the probiotic intervention. Most of the reported AEs were related to maternal gastrointestinal health, and most of them occurred during the third trimester of gestation. They included nausea, abdominal cramping, flatulence, and headache. However, similar side effects have been reported after administration of probiotics to non-pregnant populations [1]. On the other hand, these gastrointestinal symptoms are relatively common during pregnancy even in women not consuming probiotics [50]. It has also been suggested that such minor problems might be attributed to the fact that many of the products included in the reviews and meta-analysis contained not only probiotics but, also, prebiotics, plant extracts, and other ingredients [33]. In addition, one study found an increased risk of changes in stool consistency and of vaginal discharge [51] but such findings were not observed in a subsequent study using the same strains [52].

In parallel, another systematic review and meta-analysis evaluated perinatal outcomes in pregnant women that received probiotic supplements in the frame of randomized controlled trials [53]. Across the 25 trials that were meta-analyzed, the reporting of perinatal outcomes was highly inconsistent, and the only data reported in at least 50% of the studies were gestational age at birth, cesarean section, and birth weight. Overall, probiotic administration of probiotics had neither a positive nor negative impact on these perinatal outcomes. Previously, a comprehensive review of 37 studies involving the use of prenatal probiotics from 1990 to 2011 also found relevant inconsistencies in the reporting of perinatal clinical outcomes. However, no severe AEs were reported among the reviewed studies, suggesting that the different tested strains were safe and well tolerated [54]. 

Although at much lower extent than oral probiotics, vaginal probiotics may also be used during pregnancy to try to minimize changes in the vaginal secretion or the duration and/or symptoms of vaginal infections [55]. Studies on vaginal probiotics have an even higher frequency of safety reporting inconsistencies or lack of safety data compared to oral probiotics. Nevertheless, some studies reported a good tolerance and no adverse effects derived from the probiotic intervention, even in women at risk of preterm delivery [56]. The European trend to encourage companies to reposition vaginal probiotics as drugs may help to overcome this situation in the future [57].

### 4.2. Probiotics for the Eradication of Vaginal and Rectal Colonization by Group B Streptococci

Rectal and vaginal colonization by *Streptococcus agalactiae* (Group B Streptococci, GBS) during pregnancy has the potential for vertical mother-to-infant transmission, and, in some cases, can lead to neonatal early-onset sepsis (EOS), a condition with a high morbidity and mortality risk [58,59,60]. Two main approaches (risk-based and screening-based protocols) are currently followed in Western countries to try to avoid such transmission. Both approaches involve intrapartum antibiotic prophylaxis (IAP) to women that display a risk factor or that are positive after recto-vaginal GBS screening at week 35–38 of pregnancy, respectively. However, IAP-related strategies face some drawbacks and limitations since they do not prevent GBS-related abortions, stillbirths, and preterm births [61], do not guarantee GBS eradication, may increase antibiotic resistance among GBS and other pathogenic bacteria [62,63,64,65], and may have a negative impact on the acquisition and development of the infant microbiota [66,67,68,69,70], a fact that may have a lasting impact on health [71,72].

In this frame, the administration of some target-specific probiotic strains during the third trimester of pregnancy has been associated with a variable impact on the GBS recto-vaginal colonization, from no effect to a notable decrease of the colonization rates at 35–38 weeks [73,74,75,76,77,78,79,80,81]. However, all these probiotic interventions have a common feature: no adverse events associated with probiotic intake were reported. Two of them found a decrease in gastrointestinal symptoms in the probiotic arm compared to the placebo group [73,74]. On the other hand, GBS were eradicated from 70% of GBS-positive pregnant women in one of the studies [78]. Such reduction in the exposure of pregnant women and their infants to GBS and to IAP may be considered, by itself, as a beneficial safety-related outcome.

### 4.3. Prevention and Management of Gestational Diabetes Mellitus (GDM) and Hypertensive Disorders

As stated above, GDM is one of the most relevant complications during pregnancy since it may imply negative short and long-term outcomes for the mother–infant pair. Although several interventions (physical activity, diet, and/or medication) may be advisable to prevent GDM and restrict gestational weight gain, particularly among overweight and obese pregnant women, their actual efficacy is relatively limited [82]. In fact, the authors of a comparison of different strategies to prevent GDM concluded that “none of the interventions could offer remarkable benefit for GDM prevention” [82].

In this context, it is not surprising that probiotics were considered a promising approach to reduce GDM rates after the publication in 2010 of the results of a randomized, double-blind placebo-controlled trial showing that the GDM frequency was much lower among women receiving probiotics (*Lacticaseibacillus rhamnosus* GG and *Bifidobacterium animalis* ssp. *lactis* Bb12, 1 × 10^9^ colony forming units (CFU) of each strain per day, from week 20 to delivery) and diet counselling (13%) than among those receiving a placebo and diet counselling (36%, *p* = 0.003). In fact, the GDM rate of the latter group was similar to that of control women who did not receive either the probiotic or diet counselling (34%) [83]. In addition, no adverse events, including no differences in the duration of pregnancies and in infant postnatal growth rates, were reported in mothers or children of the probiotic group. Previous results from the same trial had shown that dietary counselling together with probiotics improved blood glucose control even in normoglycaemic pregnant women, providing a mean for the prevention and management of glucose disorders [84]. A Cochrane systematic review on probiotics for preventing GDM was dedicated exclusively to this trial, reaching the same conclusions [85].

The work of Luoto et al. [83] has been followed by other probiotic interventions targeting GDM rates and/or other metabolic outcomes of pregnancy. The first systematic review on probiotics and GDM that included more than one trial (one Norwegian prospective cohort study [86], one Finnish randomized trial resulting in three publications [83,84,87] and an Iranian randomized trial which results were also published in three different articles [88,89]) concluded that, despite the variety of strains, dosages and outcomes, these products seemed to be safe and reduced maternal fasting glucose, GDM, and pre-eclampsia rates and levels of the C-reactive protein [90]. It also concluded that more randomized controlled trials were urgently required, particularly among overweight and obese pregnant women since they are at high risk of metabolic and hypertensive disorders. Paradoxically, at present, there are more than 110 reviews, systematic reviews, and meta-analyses available in PubMed (29 November 2024) which have repeatedly evaluated the outcomes of a combination of up to only 15 original trials or prospective studies.

Overall, almost all the meta-analysis published up to the date agree that the administration of probiotics during pregnancy is safe and may have the potential to improve glucose metabolism and reduce GDM rates [91,92,93]. Still, they also highlight the need for further well-designed trials, involving a much higher number of pregnant women and with a proper and clear evaluation of safety and efficacy [94]. It is interesting to note that, among the 15 articles analyzed in the meta-analysis that include the highest number of studies so far [91], 11 studies had been performed in Iran by the same group and, therefore, may not be representative of pregnant women in other geographical, environmental, and socio-economical settings [95]. This problem is shared by most of the meta-analysis since, as another example, eight of these Iranian articles were included among the eleven studies included in other meta-analysis [96].

No adverse effects have been found in a meta-analysis assessing the impact of probiotics on pregnant women already diagnosed with GDM, while this intervention resulted in a significant reduction in insulin resistance [97]. However, again, three out of the four articles included in this meta-analysis were published by the Iranian group. It has been also reported that probiotic supplements in women with GDM may decrease neonatal birth weight [98]. High birth weight is a frequent complication in pregnant women with this condition. In contrast, another meta-analysis found no evidence to conclude that the administration of probiotics to pregnant women with GDM or pregnant women who are overweight may control newborn birth weight [99]. Interestingly, such apparently conflicting conclusions arose from two meta-analysis that shared most of the analyzed studies.

A Cochrane systematic review on the usefulness and safety of probiotics for preventing GDM published in 2021 [100] introduced some controversy in the, until then, general agreement that the use of probiotics during pregnancy was, in the worst of the cases, a safe practice. The conclusion of this review was that while there was not a clear beneficial effect of probiotics on the risk of GDM, the risk of preeclampsia increased with probiotic intake (9.2% in the probiotic group versus 4.9% in the placebo group, *p* = 0.09) and, therefore, the authors urged caution in the use of probiotics during pregnancy. Surprisingly, the conclusion that probiotics increased the risk of preeclampsia was based in only one of the seven studies included in the review, precisely the only one that had been authored by the same authors of the review [101]; in contrast, the remaining six studies supported that probiotics were safe during pregnancy [84,102,103,104,105,106]. This conflicting situation deserves a more detailed comment on the results obtained by the different studies included in the controversial review.

As already stated, the trial of Laitinen et al. [84] found that the probiotic strains used in their intervention (*L. rhamnosus* GG and *B. animalis* ssp. *lactis* Bb12) were safe and useful for the prevention and treatment of glucose disorders during pregnancy. Three additional publications derived from the same trial confirmed safety for mothers and infants and efficacy for reducing the frequency of GDM [83,84,107]. Aaltonen et al. [107] demonstrated that, in comparison with two placebo groups, the simultaneous administration of these two strains did not affect the values of systolic and diastolic blood pressure in any of the three trimesters of gestation, which disagrees with an increased risk of preeclampsia. It is worth mentioning that the strains, posology, and period of treatment of the trial of Callaway et al. [101] were the same that those employed in the Finnish trial [83,84,86,107]. In addition, the trial of Callaway et al. [101] contains some apparent internal contradictions: the authors found that probiotics increased the risk of preeclampsia, but this practice had no negative impact in “gestational hypertension”, in “hypertensive disorders of pregnancy”, or in the values of systolic and diastolic blood pressure at 28 or 36 weeks of gestation. In the same trial, it was found that the administration of probiotics decreased the rates of both “excessive weight gain” and “small for gestational age” when compared with the placebo arm.

The second trial included in the review of Davidson et al. [100] involved the administration of *Ligilactobacillus salivarius* UCC118 to obese pregnant women and did not report adverse effects related to probiotic intake [102]. In this study, there was no difference in the rate of preeclampsia between the probiotic and the placebo arms. A complementary part of the same trial also failed in finding differences in preeclampsia rates between both the probiotic and placebo groups [108]. The third trial in the review of Davidson et al. [100] reported that probiotic supplementation (*Lactobacillus acidophilus*, *Lacticaseibacillus casei*, *Bifidobacterium bifidum*; one strain of each species) in the first half of pregnancy had beneficial effects on inflammation and insulin and lipid metabolism and, again, no adverse effects were found [103]. The fourth trial [104] showed that the administration of a probiotic strain (*L. rhamnosus* HN001), from week 14 to week 16 of pregnancy, was safe and may be beneficial in reducing GDM prevalence, especially in the case of older women for pregnancy. Finally, the two remaining trials found that the respective probiotics tested in high-risk obese women (*L. rhamnosus* GG and *B. lactis* Bb12 [105]; *L. rhamnosus* HN001 and *B. animalis* ssp. *lactis* 420 [106]) were safe and well tolerated although failed in lowering the risk of GDM [103,104]. The trial of Okesene-Gafa et al. [104] used the same strains and at the same dose that were in the trials of Laitinen et al. [84] and Calloway et al. [101] although the probiotic intervention started later (week 28). In conclusion, it is difficult to blame probiotics of increasing preeclampsia rates based on the low-level evidence of a single trial, particularly when the rest of the trials have failed to find such association. Two more recent systematic reviews and meta-analyses specifically evaluated the impact of probiotics on the incidence of preeclampsia among pregnant women with GDM, and they found no effect on the incidence of preeclampsia in this at-risk population when compared to the placebo groups of the respective trials [109,110].

The fact that most studies have revealed, so far, that ingestion of probiotics during pregnancy and in immunocompromised adults is usually safe [111] does not mean that risk is absent. Safety and efficacy of most of the OTC probiotics ingested by pregnant women have not been previously tested in clinical trials. It has been reported that probiotics may be a cause of sepsis, liver abscess, or endocarditis in vulnerable adults with hypertension and/or diabetes [112,113,114,115,116,117,118,119]. In addition, liver abscess and *Lactobacillus* bacteremia have also been reported for *L. rhamnosus* GG [120,121,122], one of the strains that was administered to pregnant women in some of the trials cited above [83,84,87,105,107], including the one that raised the alarm of an increased risk of preeclampsia in the probiotic group [101].

## 5. Safety of Probiotics During Lactation

A variety of microbes inhabits the mammary ecosystem during lactation and, consequently, human milk contains a site-specific microbiota, which is one of the main routes of vertical mother-to-infant transmission of the first gut colonizers [24]. However, milk dysbiosis states, which are characterized by a drastic reduction in bacterial diversity, a notable enrichment in some aerotolerant bacteria, mainly of the genera *Staphylococcus* and *Streptococcus*, and a depletion in obligate anaerobes [22,123,124], may occur. These dysbiosis states may eventually lead to mastitis, a condition that represents the major medical reason for an undesired weaning.

Empiric antibiotic therapy is the conventional etiological treatment for mastitis. However, mastitis-causing bacteria can display several resistance mechanisms, including the ability to form thick biofilms inside the lactiferous ducts and the presence of some genes providing either intrinsic or transmissible antibiotic resistance. Therefore, the rates of successful treatment of mastitis with antibiotherapy are relatively low. Additionally, this practice negatively modify the milk microbiota and may impair the acquisition of maternal microbes through breastfeeding [125]. In this frame, strategies for the management of mastitis by the selection and administration of target-specific probiotic strains have been developed [22].

A few specific human milk-derived strains, mainly *L. salivarius* CECT5713, *L. salivarius* PS2 and *Limosilactobacillus fermentum* CECT5716, have been tested in clinical trials to treat or prevent acute and subacute mastitis during late pregnancy and/or the lactation period [126,127,128,129,130,131,132]. The results indicate that they were efficient, safe, and well tolerated since only a few cases of flatulence were reported and all of them were associated with the ingestion of *L. fermentum* CECT5716 [127]. In fact, Arroyo et al. [127] showed that the treatment with either *L. salivarius* CECT5713 or *L. fermentum* CECT5716 was not only significantly more efficient than antibiotherapy but, also, safer. While 9% of the women who received antibiotics developed vaginal candidiasis, this negative therapy-associated effect was not observed in any of the women that received one of these two probiotic strains. Overall, the above cited trials led to a significant reduction in the milk concentration of the mastitis-causing bacteria and, also, of the residues of some drugs (mainly anti-inflammatory and analgesic drugs) [133]. Therefore, the administration of these probiotic strains to women increased the safety of their infants. Two recent systematic reviews and meta-analyses, providing information about the strains and posology, support the efficacy of such strategy [134,135]. The conclusion of Alemu et al. [135], after analyzing randomized controlled trials involving the administration of maternal probiotic on breast milk and infant gut microbiome and health, was that “maternal probiotic supplements effectively orchestrate the breast milk and infant gut microbiome with a wide range of clinical benefits and safety”.

## 6. Safety of Probiotics Targeting Infertility

As previously stated, vaginal lactobacilli may play relevant reproductive functions, including a fine hormonal and immunological tuning of the reproductive ecosystem during the fertility window of a menstrual cycle. Recently, it has been shown that the administration of two *L. salivarius* strains to women with reproductive failure (either repetitive abortion or infertility without a known cause, and concomitant with low concentrations of vaginal lactobacilli) led to a significant increase in the rate of successful pregnancies [136,137]. Interestingly, the strains were able to increase the vaginal levels of vascular endothelial growth factor (VEGF) and transforming growth factor β1 and β2 (TGF-β1, TGF-β2) in those women who achieved a term pregnancy. The expression of these growth factors is highly regulated and coordinated during the early implantation stages, a fact that highlights their joint relevance for a successful pregnancy [138]. VEGF is a glycoprotein with a critical role for embryogenesis and embryo implantation because of its function as regulator of the vasculogenesis, angiogenesis, and vascular functions in the human endometrium [139]. TGF-β1 and β2 play well-known roles in the induction of mucosal immune-tolerance, a process that is essential for embryo tolerance [140]. Both trials showed that the strains were safe and well tolerated.

There have been some other oral or intravaginal probiotic trials aimed at increasing fertility rates during assisted-reproduction therapies with variable outcomes. None of them described any negative safety-related issue but it is not clear if it was because of a lack of AEs or because they were not assessed during the trial [141,142,143,144,145].

## 7. Recommendations

Pregnancy involves several adaptations in a woman’s body, which can sometimes lead to some symptoms or complications. Probiotics are often used to address these issues due to their general perception of safety. However, while probiotics may have the potential to interfere or counteract these physiological adaptations, this may derive in an unexpected negative effect for which, most probably, the probiotic will not be blamed.

AEs derived from a probiotic intake exist and expanding their use to vulnerable populations, such as pregnant or lactating women (a practice that may also affect their fetuses and infants), require well-informed recommendations by health professionals [2]. Probiotic trials (or, at least, part of them) specifically designed to evaluate safety are scarce, and safety concerns may be related to the strain(s), the posology, the state of each treated person (intended use), and the quality of the product [146,147].

The safety of probiotics should be based on in silico, in vitro, and in vivo studies. A thoughtful safety assessment should monitor acute and long-term risks. General recommendations about them and to guide the medical and scientific communities about probiotic safety have been already proposed [2,148]. Safety assessment may pay special attention to the target population, particularly if it can be considered vulnerable, including extra monitoring by well-informed practitioners. Whole genome sequencing of the strain(s) included in a probiotic product seems an essential requirement, since this enables the detection of some safety-related traits, including deleterious metabolic activities, antimicrobial resistance, toxin production, or genetic stability [2,149]. In vitro characterization of the strain(s) (e.g., antibiotic resistance phenotype) is also crucial but, again, such an assessment must have in account the intended use since, as an example, probiotic strains may interfere with estrogen metabolism, a process that may be relevant during pregnancy [150]. Finally, the reporting of adverse effects in randomized controlled trials have a paramount importance in safety assessment and should follow well-respected guidelines, such as updated Consolidated Standards of Reporting Trials (CONSORT) statements for the complete and detailed reporting of harms.

## 8. Conclusions

In conclusion, trials in which probiotic strains have been provided to pregnant or lactating women are scarce although, in most of them, their administration was well tolerated and safe even by obese women with GDM. However, one study has reported an increased risk of preeclampsia when probiotics were administered to overweight and obese women [101]. Although this study showed some contradictory results regarding preeclampsia markers and provided opposite results to those of other trials in which pregnant women received the same strains [83,84,87,105,107], such a risk should be considered in practice. Consequently, the administration of probiotics to obese pregnant women should be monitored to detect any increased risk of preeclampsia. Anyway, health professionals attending pregnant or lactating women should be aware of any probiotic that they may be taking during such stages, to follow up their actual efficacy, to ensure their safety, and to report any potential AE derived from probiotic intake that, otherwise, may remain unnoticed. It is obvious that absolute safety does not exist, neither for probiotics nor for drugs or foods, but the systematic collection of such information may allow for the improvement of our knowledge on the risk-to-benefit ratio of using probiotics and to select the best options in every case when they may be useful on a risk-benefit basis.

**Figure 2:**
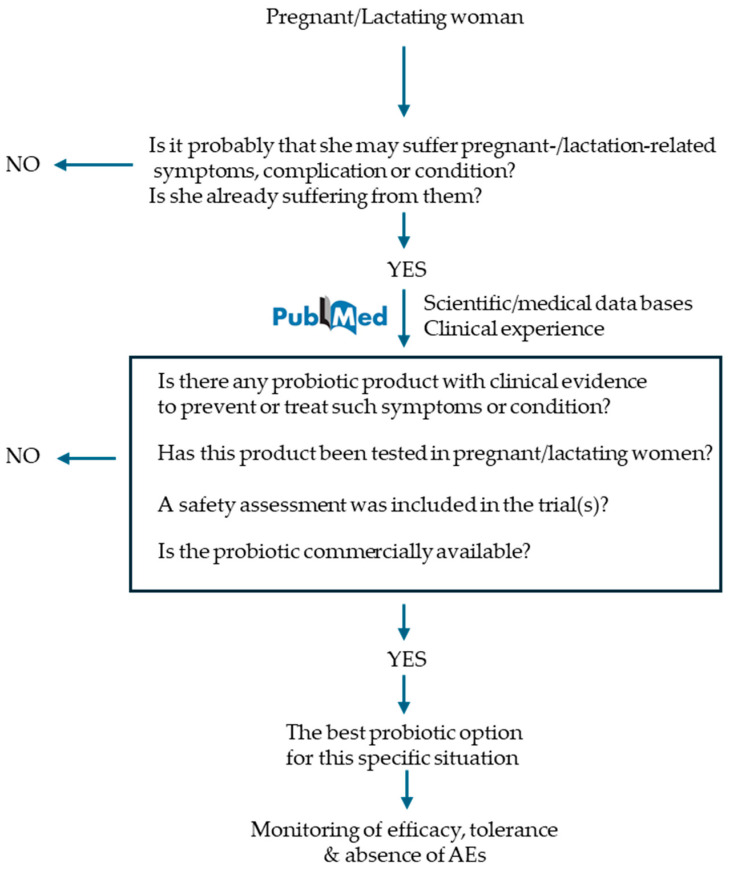
A proposal of a decision-support system for health professionals when deciding if it is worth prescribing a probiotic product to pregnant or lactating women and which product may be better in each case.

Health professionals should recommend probiotics based on their own clinical experience and the guidelines provided by medical associations in each country but, also, on scientific evidence, opting for those products that have been designed for the specific target or condition that a pregnant or lactating woman may be experiencing or at high risk of, and which efficacy and safety has already been convincingly tested in such populations (Figure 2).

## Data Availability

No new data were created or analyzed in this study. Data sharing is not applicable to this article.
